# A rare case: Pure Sertoli cell tumor uncovered in atrophic ovary during postmenopausal vault prolapse evaluation

**DOI:** 10.18632/oncoscience.619

**Published:** 2025-05-22

**Authors:** Naina Kumar, Ashutosh Rath, Nireesha Bukke, Pooja T. Rathod, Jarathi Aparna

**Affiliations:** ^1^Department of Obstetrics and Gynaecology, All India Institute of Medical Sciences, Bibinagar, Hyderabad 508126, Telangana, India; ^2^Department of Pathology, All India Institute of Medical Sciences, Bibinagar, Hyderabad 508126, Telangana, India

**Keywords:** ovary, postmenopausal, sacrocolpopexy, Sertoli cell tumor, vault prolapse

## Abstract

Introduction: Sertoli-Leydig cell tumors (SLCTs) are rare ovarian neoplasms, accounting for less than 0.2% of all primary ovarian tumors. Among these, pure Sertoli cell tumors (SCTs) are exceptionally rare, comprising only 4% of Sertoli-stromal tumors. While SCTs are more commonly observed in young women, they can occur across all age groups. They are often associated with estrogen or progesterone production, whereas testosterone production is extremely uncommon.

Case Report: A 70-year-old postmenopausal woman with a history of hysterectomy 20 years ago presented with complaints of a vaginal bulge and reduced urine output for 4–5 months. She had a prior diagnosis of periampullary carcinoma (pT1aN0), treated with surgery and adjuvant chemotherapy using gemcitabine. Clinical examination revealed a fair general condition and unremarkable abdominal findings. Per speculum examination showed vault prolapse with third-degree cystocele and minimal rectocele but no signs of stress urinary incontinence. The patient underwent abdominal sacrocolpopexy for vault prolapse. During surgery, both atrophic ovaries and fallopian tubes were identified and removed. Histopathological examination confirmed a Stage Ia pure SCT in the right ovary, with no malignancy in the left ovary or fallopian tubes.

Conclusion: The incidental discovery of a pure SCT in an atrophic ovary during pelvic surgery in a postmenopausal woman is exceedingly rare. This case highlights the importance of meticulous intraoperative inspection and histopathological evaluation, even in asymptomatic atrophic ovaries.

## INTRODUCTION

Sertoli-Leydig cell tumors (SLCTs) are a rare and diverse group of ovarian neoplasms classified under the sex cord-stromal category of tumors. They account for less than 0.2% of all primary ovarian neoplasms [1]. Within this category, pure Sertoli cell tumors (SCTs) are even rarer, comprising approximately 4% of all Sertoli-stromal cell tumors of the ovary [2]. Pure SCTs lack a Leydig cell component and are devoid of the immature neoplastic stroma typically seen in SLCTs [3]. The exact mechanism behind the development of ovarian SCTs remains unclear. These tumors are believed to arise from ovarian cells that retain the ability to differentiate both in appearance and function from Sertoli cells [4]. Recent studies have identified a strong association between SLCTs and germline mutations in *DICER1*, while *FOXL2* mutations are rarely observed [5, 6].

Pure SCTs of the ovary typically occur in women of reproductive age, with an average onset of around 30 years [4]. Less than 10% of cases are diagnosed before menarche or after menopause [2]. SLCTs are characterized by the presence of Sertoli and Leydig cells and are often testosterone-producing. Consequently, approximately 40–50% of women with SLCTs exhibit signs of androgen excess, such as virilization. The remaining cases may present as non-functional tumors or estrogen-secreting tumors, typically identified during evaluations for amenorrhea or infertility [7, 8].

Given the rarity of pure Sertoli cell tumors, especially when incidentally found in postmenopausal women, this case is being reported.

## CASE REPORT

A 70-year-old postmenopausal woman, who had undergone a hysterectomy 20 years prior, presented to the Obstetrics and Gynecology outpatient department with complaints of something coming out of the vaginal and decreased urine flow over the past 4–5 months. She denied symptoms of stress urinary incontinence, dysuria, burning micturition, abdominal distension, vomiting, loss of appetite, or postmenopausal bleeding. Additionally, there were no signs of hyperandrogenism.

Her medical history included Whipple’s surgery six years ago for periampullary carcinoma (pT1aN0), followed by adjuvant chemotherapy with gemcitabine (1000 mg/m² IV infusion weekly for 7 weeks, a week of rest, and then weekly infusions for 3 weeks in each 28-day cycle). The postoperative and post-chemotherapy period was uneventful.

On general examination, the patient had a BMI of 24.5 kg/m², was well-oriented, and had a blood pressure of 130/80 mmHg. Abdominal examination revealed a soft, non-tender abdomen with no palpable masses or free fluid. On local examination, atrophic labia majora and minora were noted without abnormalities. Per speculum examination revealed vault prolapse with third-degree cystocele and minimal rectocele but no signs of stress urinary incontinence on coughing or straining. The rest of the vagina appeared healthy. A Pap smear of the vaginal vault was negative for intraepithelial lesions or malignancy. Bimanual examination confirmed free bilateral fornices and a healthy vault.

Transvaginal ultrasonography showed bilateral atrophic ovaries with no other abnormalities.

The patient was admitted for abdominal sacrocolpopexy to address vault prolapse. Preoperative investigations, including complete blood counts (Hb 13.1 g/dL, total leukocyte count 6,400/mm³, platelet count 240,000/mm³), liver and renal function tests, thyroid function tests, and fasting (98 mg/dL) and postprandial blood sugars (128 mg/dL), were all within normal limits.

Intraoperatively, the vaginal vault was densely adherent to the rectum posteriorly and bladder anteriorly, with bilateral atrophic ovaries and fallopian tubes. The ovaries and fallopian tubes were removed by clamping the infundibulopelvic ligaments. The vault was dissected sharply from the bladder and bowel and suspended retroperitoneally using Mersilene tape, which was anchored to the periosteum of the sacral promontory and sacral anterior longitudinal ligament. The cystocele was repaired abdominally. The peritoneum, omentum, and bowel appeared grossly healthy, with no ascites or visible deposits.

**Figure 1:**
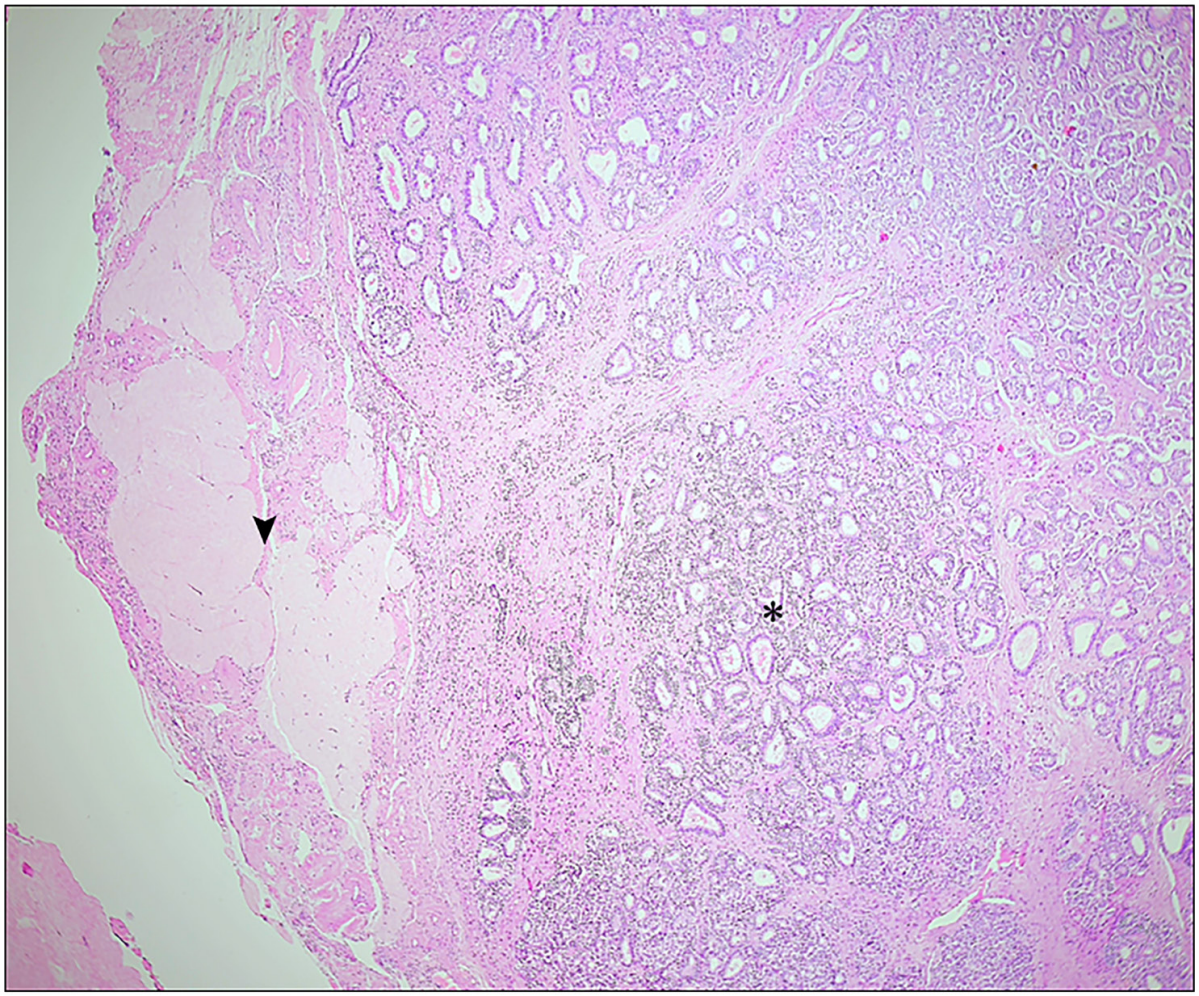
Pure Sertoli cell tumor in the right ovary (*) with adjacent ovarian tissue (Arrow) (4×).

**Figure 2:**
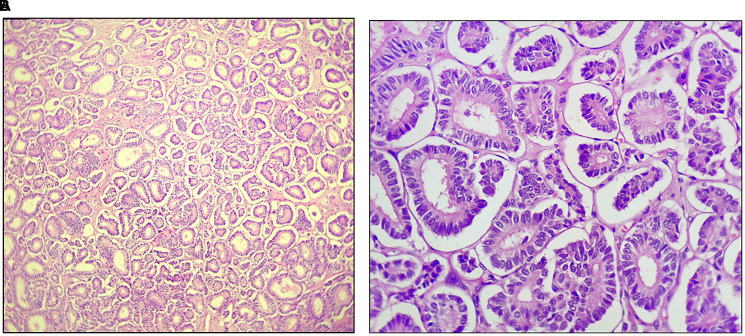
Tubules of tumor cells in Sertoli cell tumor (10×; 40×).

On gross appearance, the right ovary measured 3 × 2 × 1 cm, and on the cut section had a grey-white nodule measuring 1.5 × 1 × 1 cm. The left ovary measured 3 × 1.5 × 1 cm with an unremarkable cut surface. Bilateral fallopian tubes were unremarkable. Microscopically, the right ovary showed a well-delineated tumor composed of tubules separated by fibrous septa. The tumor cells, cuboidal to columnar in shape, exhibited round to oval nuclei with evenly distributed chromatin, focal small nucleoli, and nuclear grooves. Adjacent ovarian tissue was unremarkable. The left ovary and fallopian tubes showed no significant abnormalities, apart from dystrophic calcification in blood vessels (Figures 1 and 2A, 2B). Histopathological findings confirmed a stage Ia pure Sertoli cell tumor (SCT) in the right ovary, with no evidence of malignancy in the left ovary or fallopian tubes. Given the early-stage diagnosis, adjuvant chemotherapy was not required.

In this case, the patient was incidentally diagnosed on histopathological examination with stage Ia pure SCT and did not require adjuvant chemotherapy. She tolerated the surgery well and had an uneventful postoperative recovery. The sutures were removed, and she was discharged on the eighth postoperative day in stable condition. Currently, she remains under regular follow-up and is doing well, with no reported complaints.

## DISCUSSION

SLCTs are rare, predominantly unilateral ovarian tumors, with only 1.5% presenting bilaterally, and are often confined to the ovary at diagnosis. Histologically, they consist of Sertoli and Leydig cells in varying proportions and are classified as well-differentiated, intermediately differentiated, or undifferentiated [9]. Pure SCTs, are exceptionally rare and are characterized by the absence of Leydig cells and immature stroma, setting them apart from SLCTs, which contain both components [4]. Microscopically, SCTs feature round or elongated tubules lined by cuboidal or columnar cells, typically without atypical features or significant mitotic activity. Differentiation ranges from well- to poorly differentiated forms [10]. The immunohistochemical panel used for diagnosing SCTs typically includes markers such as inhibin, SF1, CD99, and calretinin, which play a crucial role in establishing a definitive diagnosis. In contrast, SCTs typically yield negative results for markers like epithelial membrane antigen (EMA), CK7, PAX8, GATA3, and chromogranin [11].

SCTs predominantly produce estrogen, with progesterone or testosterone production being exceptionally rare. Elevated estrogen levels can lead to menstrual irregularities, postmenopausal bleeding, or endometrial hyperplasia. In contrast, progesterone production may cause decidualization of the endometrium or peritoneum, while increased testosterone levels are associated with symptoms such as amenorrhea or virilization [4]. When a genetic association is present, SCTs are most commonly linked to Peutz-Jeghers syndrome, which is caused by mutations in the *STK11/LKB1* gene [3]. Due to their rarity and often non-specific clinical presentation, ovarian SCTs are challenging to diagnose. A definitive diagnosis is typically established post-surgery through histological examination of the tumor [4].

SLCTs are rare ovarian malignancies with low recurrence rates and generally favorable outcomes compared to malignant epithelial ovarian tumors. Surgical resection is the cornerstone of treatment, with fertility-sparing conservative surgery being the preferred approach for younger patients to preserve reproductive potential [12]. Recent studies suggest that lymphadenectomy has no significant effect on tumor recurrence rates or disease-free survival in patients with malignant sex-cord stromal tumors (SCSTs) [13–15]. Given the low incidence of lymph node metastasis (approximately 0–4.5%) in early-stage SCSTs, routine lymphadenectomy is not recommended during initial staging surgery [16, 17]. Additionally, most ovarian Sertoli cell tumors (SCTs) are diagnosed at stage I and typically follow a non-aggressive clinical course [3]. Current guidelines advocate for lymphadenectomy only in cases where imaging or intraoperative findings suggest lymph node involvement [4, 18].

For stage Ia tumors, conservative surgery is a safe and effective option, especially for women of reproductive age. However, adjuvant chemotherapy is recommended for stage Ia cases with poor prognostic factors, such as poor differentiation, a retiform pattern, or heterologous elements. In stage Ic1 and more advanced stages, radical surgery combined with adjuvant chemotherapy is advised. The most commonly used chemotherapy regimen includes a combination of bleomycin, etoposide, and cisplatin (BEP) at a dosage of bleomycin 30 units per week on days 1, 8, and 15; etoposide 100 mg/m^2^/day daily for days 1–5; and cisplatin 20 mg/m^2^/day daily for days 1–5 for 3–4 cycles [8].

Patients with stage I, grade 1–2 SLCTs generally have an excellent prognosis and typically do not require adjuvant chemotherapy. In contrast, grade 3 SLCTs are associated with a higher risk of adverse outcomes, warranting more aggressive surgical management and rigorous post-treatment surveillance to monitor for recurrence or progression [19]. Current guidelines recommend adjuvant chemotherapy for SLCTs with heterologous elements, poor differentiation, or classified as FIGO stages IB through IV [6].

SLCTs generally have a favorable prognosis, with survival outcomes varying by differentiation and stage [2, 12, 20]. The 5-year survival rate is 100% for well-differentiated tumors, approximately 80% for moderately and poorly differentiated cases, and 95% for stage I disease. However, survival is significantly lower in advanced stages, with minimal to no survival reported for stages III and IV [2].

Due to the rarity of this tumor, data on SLCT recurrence remains limited. While SLCT generally has a favorable prognosis, reported relapse rates vary widely, ranging from 0% to 33.3% [9, 21, 22]. Recurrence typically occurs early, with 95% of cases relapsing within the first five years [7, 8]. The prognosis for recurrent SLCT is poor, with a salvage rate of less than 20% for clinically malignant and recurrent disease [23]. Relapses are often unifocal or multifocal, primarily localized in the pelvis or abdomen [7]. A multi-modal treatment strategy, combining surgery with chemotherapy, currently offers the most effective approach for managing relapses. However, the optimal chemotherapeutic regimen remains undefined and requires further investigation [7]. Therefore, regular follow-up is essential, with evaluations every three months during the first year, every six months in the second year, and annually thereafter [24].

## CONCLUSIONS

The incidental discovery of a pure SCT in an atrophic ovary of a post-hysterectomized postmenopausal woman undergoing vault prolapse surgery is extremely rare. This case emphasizes the importance of meticulous intraoperative inspection and histopathological evaluation, even in atrophic ovaries, as such tumors can be present without obvious clinical symptoms. The rarity of pure SCTs in this context highlights the need for heightened awareness and thorough investigation during pelvic surgeries in postmenopausal women. Given the potential for hormonal activity and the rare risk of malignant transformation, early identification is essential for optimal management and long-term surveillance.
